# Minimally Invasive Colorectal Surgery Under General Versus Neuraxial Anesthesia: A Retrospective Propensity-Score-Matched Analysis

**DOI:** 10.3390/jcm14217684

**Published:** 2025-10-29

**Authors:** Carlo Ferrari, Jacopo Crippa, Davide Vailati, Benedetta Basta, Salvatore Barbaro, Michele Colasuonno, Roberto Santalucia, Carmelo Magistro

**Affiliations:** 1 Division of General Surgery, Vizzolo Predabissi Hospital, ASST Melegnano e Martesana, via Pandina 1, Vizzolo Predabissi, 20077 Milan, Italy; carlo.ferrari@asst-melegnano-martesana.it (C.F.); salvatore.barbaro@asst-melegnano-martesana.it (S.B.); michele.colasuonno@asst-melegnano-martesana.it (M.C.); roberto.santalucia@asst-melegnano-martesana.it (R.S.); carmelo.magistro@asst-melegnano-martesana.it (C.M.); 2 Division of Anesthesiology and Intensive Care, Vizzolo Predabissi Hospital, ASST Melegnano e Martesana, via Pandina 1, Vizzolo Predabissi, 20077 Milan, Italy; davide.vailati@asst-melegnano-martesana.it (D.V.); benedetta.basta@asst-melegnano-martesana.it (B.B.)

**Keywords:** colorectal surgery, laparoscopic surgery, neuraxial anesthesia, minimally-invasive surgery, colorectal cancer, diverticular disease, combined spinal–epidural

## Abstract

**Background**: Neuraxial anesthesia, delivered as a combined spinal–epidural without intubation or neuromuscular blockade, is a promising alternative for patients undergoing minimally invasive colorectal surgery. Evidence is limited to case series, with no cohort studies available. **Methods**: This is a retrospective analysis of consecutive patients undergoing minimally invasive colorectal surgery for both benign and malignant disease at a single institution, between October 2022 and October 2024. Patients were divided by the type of anesthesia. Propensity score matching was performed to reduce confounding bias. Outcomes assessed included anesthesiologic preparation time, duration of surgery, intraoperative features, intensive care unit admission, length of hospital stay, and 90-day postoperative complications, including anastomotic leak and readmission rates. **Results**: Thirty-two patients (40.5%) received neuraxial anesthesia and forty-seven (59.5%) received general anesthesia. No conversions from neuraxial to general anesthesia occurred. After matching, anesthesia preparation time was longer in the neuraxial group (42.5 vs. 30 min, *p* = 0.011), while operative time was significantly shorter (181 vs. 231 min, *p* = 0.002). Length of stay, postoperative complications, including leak, and readmission rates were comparable between groups. **Conclusions**: Neuraxial anesthesia may be a valid alternative to general anesthesia for minimally invasive colorectal surgery. In this single-center experience, it required longer anesthetic preparation but was associated with shorter operative times, without affecting surgical outcomes or increasing complication rates. These results support further investigation into its application in colorectal procedures.

## 1. Introduction

Traditionally, general anesthesia (GA) has been the standard for most major surgeries due to its ability to obtain sufficient myorelaxation for workspace optimization during laparoscopy and control of the patient’s airways. However, advances in neuraxial anesthesia (NA), like combined spinal–epidural anesthesia (CSE), have expanded the anesthetic options for patients undergoing both open and minimally invasive surgeries. This technique combines the rapid onset and profound block provided by spinal anesthesia with the extended duration and flexibility of epidural anesthesia, allowing for better postoperative analgesia and a reduced need for systemic opioids [1]. These advances have provided comparable surgical conditions to GA, making NA an appealing choice.

There is growing concern within the surgical community about the gap in care for patients who have a surgical indication and wish to proceed with the procedure but are advised against it following the anesthesiological evaluation due to an unfavorable risk–benefit profile. This may be driven by concerns over potential pulmonary complications, reduced functional reserve, or the risk of cognitive decline [2]. Another important consideration is patients who actively prefer to avoid GA due to potential adverse effects and the loss of control over their bodies. One of the key factors driving interest in NA is its suitability for elderly and frail patients who may be considered too high risk for GA. Additionally, it offers a viable option for those who specifically seek a GA-free surgical approach.

There has been a natural counterthought to operate on patients without the use of GA, particularly for major surgeries and laparoscopic procedures. The possibility of performing surgery without gas-based anesthesia, avoiding curarization and intubation, may seem compelling and suggests potential advantages from the patient’s perspective, paving the way for a less invasive approach.

The potential of NA in major abdominal procedures is largely unexplored, and data are needed to establish NA as a viable and safe alternative to GA. In 2022 we developed an institutional protocol to provide NA as an alternative option to GA for patients fulfilling specific inclusion criteria. The aim of this study is to report our initial experience of patients undergoing minimally invasive colorectal surgery for benign and malignant disease under NA, compared with a similar population undergoing GA.

## 2. Materials and Methods

This is a retrospective study of data from a prospectively collected database of patients undergoing colorectal surgery for benign or malignant disease at the General Surgery Division of the Hospital of Vizzolo Predabissi, Milan (Italy), from October 2022 to October 2024.

Inclusion criteria included the following:

- Age 18 or older;
- Surgical indication for colorectal resection for benign and malignant disease;
- Informed consent for research purposes.

Exclusion criteria were as follows:

- Open surgical procedures;
- Surgery for recurrent oncologic disease.

At our institution, NA is offered to all patients fulfilling at least one of the following inclusion criteria:

- Age 80 years or older;
- Risk factors for postoperative cognitive decline (POCD) (e.g., cognitive impairment, memory lability, mini-mental test [3] score < 8);
- Risk factors for postoperative respiratory insufficiency and/or pulmonary complications (e.g., ARISCAT score [4] > 45);
- Reduced functional reserve (e.g., clinical frailty score [5] > 4);
- Patients requesting surgery without GA.

Whenever meeting one of these criteria, patients are counselled for both GA and NA by the anesthesiology team. After counselling, patients are asked to give their consent to GA or NA and the type of anesthesia is chosen accordingly.

Approval for the study was obtained from the local Institutional Review Board (protocol number: CET 198-2023 and CET Em. 318-2025).

### 2.1. Preoperative Management

All patients undergoing colorectal surgery at our Institution adhere to a comprehensive Enhanced Recovery After Surgery (ERAS^®^) pathway, in line with the latest guidelines [6]. During the counseling session, a surgeon, anesthesiologist, dietitian, physiotherapist, and the case manager meet the patient and his/her caregivers, and the key points of the ERAS^®^ protocol are discussed, explaining the actions that will be taken by each participant in the pathway. A fifteen-day prehabilitation period is planned: physical status optimization and respiratory capacity training are achieved through interventions on modifiable risk factors (e.g., smoking and alcohol cessation) in conjunction with mild, daily physical activity (a daily 30 min walk) and respiratory training using an inspiratory exerciser, which allows patients to visualize their breathing performance and encourages deeper and longer breaths. A low-residue diet is typically recommended two weeks before surgery. As the surgery date approaches, oral immunonutrition (Impact^®^ Oral drink, Nestlé, Vevey, Switzerland) is administered, tailor-dosed to each patient’s specific nutritional status. Preoperative bowel preparation with rifaximine and metronidazole and mechanical preparation are administered to all patients.

### 2.2. Intraoperative Management

A target control infusion (TCI) GA is performed using Schnider’s model for propofol [7] and Minto’s model for remifentanil [8]. Mechanical ventilation is set with tidal volumes (TV) between 6 and 8 mL/kg. Initial positive end-expiratory pressure (PEEP) is set at 5 cmH_2_O; values are then individualized for each patient based on respiratory mechanical measurement (best PEEP). Fraction of inspired oxygen (FiO_2_) is generally set between 40 and 50% in order to maintain a peripheral oxygen saturation (SpO_2_) equal to or above 96%. An epidural catheter is utilized for postoperative analgesia in all patients.

NA is achieved by means of a CSE anesthesia. Spinal anesthesia is performed between T8 and T9, T9 and T10, or T10 and T11, depending on the scheduled procedure. The level of anesthesia is always tested using a skin prick test, with the goal of reaching at least the T2 level. A combination of 2 mL of 0.25% levobupivacaine (10 mg) and 8 mcg dexmedetomidine is injected intrathecally. In case of surgeries involving the sacral plexus, additional saddle anesthesia is performed through an L3–L4 spinal anesthesia, injecting 2 mL of 0.25% hyperbaric bupivacaine and 4 mcg dexmedetomidine. The epidural catheter is inserted between T7 and T8 or T8 and T9, above the spinal puncture. Epidural analgesia is achieved with continuous perfusion of 0.2% ropivacaine, beginning 2 h after spinal anesthesia. Intravenous sedation is obtained by infusion of 0.1–1 mcg/kg/h of dexmedetomidine and ketamine boluses (up to 100 mg in total). GABAergic drugs are avoided, and the sedation goal is a Richmond Agitation–Sedation Scale score of 0/−2. Hemodynamic control of hypotensive episodes is achieved with prompt etilefrine boluses; when necessary, continuous infusion of norepinephrine is started, which is stopped at the end of surgery. Patients undergoing AS breathe spontaneously, with oxygen supply being provided through conventional nasal cannulas.

The laparoscopic surgical approach is offered to all the patients undergoing colorectal surgery at our institution, with limited exceptions in case of multi-organ involvement of the primary disease or anesthesiologic contraindication. All surgical procedures were performed by the same surgeon experienced in colorectal surgery. For all colic resections, pneumoperitoneum is obtained using a Veress needle at Palmer’s point. Right colectomy for cancer is performed following the complete mesocolic excision (CME) principles [9]. In left colic and low anterior rectal resections for malignant pathology, lymphadenectomy is performed at the origin of the inferior mesenteric artery (IMA), followed by high or low ligation of the IMA. The inferior mesenteric vein is dissected and divided below the pancreatic border. In case of benign diseases, such as diverticular disease, instead, the sigmoid mesocolon is incised over the IMA arch; sigmoid arteries are individualized and divided, sparing the superior rectal artery.

### 2.3. Postoperative Management

As per ERAS^®^ protocol, patients are given an oral diet on the same day of surgery and are prompted for early mobilization. The urinary catheter is removed on postoperative day (POD) 1. Patients are instructed to carry on with the preoperative respiratory exercises. Pain is assessed three times a day, based on a numeric pain rating scale ranging from 0 to 10. In patients undergoing GA, postoperative analgesia is achieved through a combination of intravenous scheduled analgesics and rescue doses. Medications include paracetamol and non-steroidal anti-inflammatory drugs (NSAIDs), with opioid derivatives as a third-line option. After NE, epidural analgesia is provided with ropivacaine infusion at 5 to 7 mL/h. In case of severe pain, patients can receive one extra bolus every 2 h. Additional postoperative analgesia is provided by administering paracetamol (1000 mg every 8 h). Epidural analgesia never exceeds 3 days to minimize the risk of complications associated with prolonged epidural catheter use.

Patients fulfill discharge criteria when the patient has tolerated at least three complete meals, flatus has passed, and the patient’s mobilization is adequate. Patients are followed up in the outpatient clinic 10 to 12 days after hospital discharge, where appropriate interventions are made to address any concerns or complaints.

Outcomes of interest included anesthesiologic set-up duration, length of surgery, length of stay (LOS), and 90-day readmission rate. All complications were assessed in-hospital and until 30 days postoperatively and graded according to the Clavien–Dindo classification. Surgical complications included ileus, anastomotic leak, postoperative need for blood transfusion, and wound infection. Anastomotic leak was defined according to the classification proposed by the International Study Group of Rectal Cancer and assessed for patients undergoing restorative surgery [10]. Postoperative ileus was defined as postoperative nil per os for more than 3 days or need for naso-gastric tube insertion. Non-surgical complications included cardiovascular and pulmonary complications, urinary tract infections, and acute kidney injury. Postoperative anemia was defined as Hemoglobin (Hb) level < 13 g/dL for men and <12 g/dL for women. Blood transfusion was administered when patients developed symptoms related to anemia or when Hb levels reached <7 g/dL (<9 g/dL in patients with a history of cardiac disease).

### 2.4. Statistical Analysis

Data were recorded in a prospectively collected database (Excel, Microsoft Corp., Redmond, WA, USA). Statistical analysis was performed by means of JMP Pro ver. 18 (SAS Institute Inc., Cary, NC, USA) and Python ver. 3.12.5 (Python Software Foundation, Beaverton, OR, USA). The descriptive statistics for the demographic and clinical characteristics of patients were expressed as mean ± standard deviation (SD) or median and Interquartile Range (Q1–Q3; IQR) for continuous variables; categorical variables were expressed as absolute frequency (with percentage). Association between categorical variables was investigated using Fisher’s exact test. The normality assumption was assessed using the Shapiro–Wilk’s test and inspected graphically through QQ plots. Student *t* test was exploited to assess mean differences; instead, when the assumptions to use a parametric test were not met, a Mann–Whitney U test was performed. Propensity score matching (PSM) was performed using R Core Team, 2024 (R Foundation for Statistical Computing, Vienna, Austria). PSM with replacement was performed involving selected control subjects (GA group) who were matched with treated subjects (NA group) based on their propensity scores, allowing the same control to be used multiple times for different treated individuals. This approach was used to reduce bias while maximizing the sample size and improving precision. Matching was performed using nearest-neighbor methods, ensuring that each treated subject was paired with the closest available control. All statistical tests were 2-sided, with a significance level set at *p* < 0.05.

## 3. Results

A total of 79 patients underwent elective laparoscopic colorectal surgery during the study period. Of these, 47 (59.5%) received GA, while 32 (40.5%) underwent NA without intubation or curarization. The most common diagnosis was malignant disease (79.7%), while 20.3% of patients had benign conditions, including 15 cases of diverticular disease and 1 case of Crohn’s disease not evident in preoperative histology.

Table 1 reports the preoperative characteristics before PSM. Patients in the NA group were older, with a significant age difference between groups [72 (61–80) vs. 83 (74–85); *p* = 0.0005]. Other baseline characteristics were similar. The most performed procedure was right colectomy with CME, occurring in 49.4% of patients. The second most common procedure was left colectomy with 29.1% cases, while 15.2% patients underwent low anterior rectal resection. Other operations were Miles operation (3.8%), transverse resection (1.3%), and total proctocolectomy (1.3%). A stoma was fashioned in 19.0% of patients. None (0%) of the 32 patients in the NA group required conversion to GA. Three patients (4.7%) were converted to open surgery, all in the GA group. Median surgery duration was 190 (165–235) minutes.

Median overall LOS was 4 (3–5) days, with a total of six (7.6%) patients readmitted within 90 days from surgery. The overall anastomotic leak rate was 3.8% (three cases), with one grade C leak (2.1%) occurring in the GA group. The overall complication rate was 26.6%, occurring in 9 (19.1%) patients in the GA group and 12 (37.5%) in the NA group. Six patients (7.6%) were readmitted within 90 days, three per group (6.4% vs. 9.4% of the GA and NA groups, respectively).

Table 2 describes the groups after PSM, with 32 patients in each group. Age [82 (72–86) vs. 83 (74–85); *p* = 0.7466] and other baseline characteristics were balanced between groups.

Table 3 presents intraoperative outcomes after PSM. The most common procedure was right colectomy with CME (65.6%). One patient in the GA group underwent transverse resection for a mid-transverse tumor, while, overall, seven (10.9%) underwent low anterior resection. No significant differences were observed in primary procedures. Anesthesia preparation time was longer in the NA group [42.5 (30–68) vs. 30 (16.2–62.2) minutes; *p* = 0.0115], while operative time was shorter [181 (157–210) vs. 231 (179–240) minutes; *p* = 0.002]. A surgical drain was used in 23.4% of cases. Additional procedures were performed in 24 patients (37.5%), including adhesiolysis (12), cholecystectomy (4), ovarian cyst removal (2), incisional hernia repair (2), ureteral stent placement (2), circumcision (1), ileocecal resection (1), hepatic segmental resection (1), ileal resection (1), and splenectomy (1). In one patient in the NA group, a splenic flexure tumor was found intraoperatively to infiltrate the pancreatic tail, requiring distal pancreatectomy and splenectomy without the need for conversion to GA.

Table 4 reports postoperative outcomes after PSM. Six patients (9.4%) required postoperative intensive care monitoring, with no difference between groups (*p* = 0.6719). Length of stay was comparable [GA: 4 (3–7) vs. NA: 5 (4–6) days; *p* = 0.6360]. The total number of anastomotic leaks was two, resulting in an overall rate of 3.1%. Both leaks (one grade A and one grade B) occurred in the NA group, representing 6.2% of patients. The grade C leak was excluded from analysis after PSM. One patient (1.3%) in the NA group died postoperatively after reoperation for bowel perforation (anastomotic leak excluded intraoperatively), with no evident iatrogenic injury identified on video review of the index operation. Another patient in the GA group died two months postoperatively from unrelated causes.

## 4. Discussion

This study offers important insights into the use of NA in minimally invasive colorectal surgery, showing that it may be considered a viable alternative to GA. LOS and overall complication rates were similar between groups. Anesthesia preparation was longer, while surgical time was shorter in the NA group.

In our study, NA was offered to a specific population meeting defined inclusion criteria. Given the potential benefits of avoiding GA, particularly in terms of preserving lung function and protecting cognitive function, these criteria identified a fragile patient group compared to the general population typically undergoing surgery with GA. To minimize confounding factors, a PSM analysis was performed. Our findings support the role of NA, showing that a carefully selected fragile population achieved outcomes comparable to those of a general population. In this study, NA was compared with GA in patients undergoing minimally invasive colorectal surgery. The analysis specifically focused on the anesthetic modality rather than “non-anesthetic” procedures, which were not within the purpose of this study.

As the population continues to age, the number of older adults undergoing surgery is expected to rise. However, today, age alone is no longer considered a reliable indicator of perioperative risk. A new concept is emerging, where frailty, a state of diminished physiological reserve beyond normal aging, has become a key predictor of poor surgical outcomes [11]. In surgical populations, frailty rates range from 10.4% to 37% in general surgery patients [12]. In this population anesthesia can increase the risk of persistent POCD [13], thus reflecting in prolonged hospital stays, lower quality of life, and raised healthcare costs [13]. A potential advantage of NA in frail patients is the need for minimal sedation during surgery, which could theoretically reduce the risk of POCD [14].

Despite mixed findings in the literature, regional anesthesia is often recommended for frail patients since it has also been linked to reduced risks of thrombolytic events, blood loss, and deep vein thrombosis in specific surgeries [15]. Additionally, it provides better postoperative pain control, improving patient comfort and reducing the likelihood of adverse cardiac events [14]. Other advantages include faster return of bowel function and better preservation of immune function after surgery, both of which are particularly important for frail patients [14].

The concept of surgery without intubation has been revisited recently, largely as a response to the challenges posed by the COVID-19 pandemic. The COVID-19 pandemic further underscored the value of NA, as anesthesiologists sought techniques that minimized the risk of viral transmission. GA typically requires endotracheal intubation, a procedure known to generate aerosols, which increases the risk of exposure to SARS-CoV-2 for healthcare providers [4]. In contrast, neuraxial techniques do not require intubation, significantly reducing the production of respiratory droplets during surgery. As a result, many healthcare systems favored regional anesthesia during the pandemic, particularly in COVID-positive or suspected patients, to protect staff from potential infection.

Most of the available studies on abdominal surgery performed with NA consist of case reports [16,17] or small case series [18,19], with only one randomized clinical trial [20] standing out among them. This imbalance in study types highlights a general lack of large-scale, high-quality research in this area. Most of the surgeries were performed using the open approach, with relatively few instances of minimally invasive techniques. The outcomes reported in these studies are often vague, broad, or inconsistently documented, with some studies failing to clearly define the results or metrics used to assess success. This lack of specificity makes it challenging to compare findings across studies or to draw meaningful conclusions about the effectiveness of techniques applied. Moreover, the statistical power of most studies is uniformly low, reflecting small sample sizes and inadequate methodological rigor, which limits the validity and generalizability of the findings. As a result, no strong conclusions or evidence-based recommendations can be made, underscoring the need for more robust and well-designed research in this field to better understand the implications of these approaches. Our study, despite the limitations of being conducted at a single center and by a single surgeon, aims to offer a methodologically sound investigation on the topic and to encourage further research.

Regional anesthesia in laparoscopy may offer numerous advantages, including faster recovery, reduced postoperative nausea and vomiting (PONV), less pain, shorter hospital stays, cost savings, improved patient satisfaction, and overall safety [21]. Our data did not univocally support these findings, as no statistically significant differences were demonstrated in the postoperative outcomes analyzed. However, the lack of significance may be attributed to the small sample size and the potential “confounding” effect of the ERAS^®^ protocol, which likely plays a more significant role in reducing the surgical stress response and promoting faster patient recovery.

The significantly shorter operative time observed in the NA group may also reflect procedural dynamics beyond physiological factors. The presence of an awake patient and adherence to a standardized perioperative protocol may have contributed to enhancing the team’s focus and coordination, fostering a heightened level of concentration and efficiency that limited unnecessary time waste during the procedure.

NA avoids common issues like sore throat, muscle pain, and airway trauma that are generally associated with GA [21]. Contrary to common beliefs, our cases have demonstrated that it is possible to achieve working pressures, table tilt, and oxygen saturation comparable to those in laparoscopic surgeries performed under GA. In our experience, simple tips and tricks can be applied to enhance patient comfort. For example, a gentle insufflation of pneumoperitoneum, never exceeding a flow of 3 L/min, and slow trocar insertion to reduce the abrupt variations in the intra-abdominal pressure, thus avoiding discomfort to the patient. The intra-abdominal pressure should be kept as stable as possible to reduce fluctuations throughout the surgery. Additionally, our practice has demonstrated that extreme positions, such as Trendelenburg’s position, are feasible if table tilting is performed slowly to avoid a falling sensation for the awake patient. These precautions help to prevent unwanted movements or contractions, ensuring optimal surgical comfort under NA. This suggests that NA can be effectively utilized for more complex procedures involving significant organ manipulation, such as colorectal surgeries, rather than being limited to minor laparoscopic surgeries. Overall, NA may offer several advantages, including reduced anesthetic exposure, avoidance of airway instrumentation, and potential modulation of the inflammatory and stress response. However, its limitations may include the need for patient cooperation and the current lack of large-scale data on rare events.

Recent evidence highlights the protective role of regional anesthesia, particularly NA, in reducing the perioperative stress response. Epidural anesthesia has been found to attenuate the endocrine and metabolic response to surgery, lowering the levels of stress markers like catecholamines and cortisol, which are typically elevated during general anesthesia [22]. This reduction in the surgical stress response positively impacts postoperative recovery, including faster restoration of gastrointestinal function, improved glucose tolerance, and a shorter hospital stay [23]. Notably, epidural anesthesia has also been shown to decrease postoperative ileus and improve pain management, which contributes to better surgical outcomes [24]. In cardiac surgery, thoracic epidural anesthesia has demonstrated a reduction in stress hormone levels and improved cardiac recovery, particularly after coronary artery bypass grafting [25]. Overall, regional anesthesia, by mitigating the trauma-related stress response, enhances recovery and reduces postoperative complications, underscoring its importance in modern surgical care. In elderly or frail patients, NA may attenuate the physiological stress response and facilitate smoother postoperative recovery, supporting its potential use in high-risk populations.

Although rare, one of the most common fears patients have about GA is the possibility of being awake and paralyzed during surgery, unable to communicate their awareness. Studies estimate the risk of accidental awareness to be about 1 to 2 in 1000 cases [26]. This undesired event can lead to severe psychological effects, including the onset of chronic post-traumatic stress disorder (PTSD) [27]. Thanks to the standardized application of NA, our team aimed to shift the paradigm from the traditional negative perception of “awareness” during anesthesia to a positive and intentional concept. In this new perspective all key players involved in the surgical procedure—surgeons, anesthesiologists, and the care team—are fully conscious of the strategy to push minimally invasive techniques to their limits. This collective awareness underscores our commitment to preserving the patient’s cognitive function by minimizing the physiological stress of anesthesia and surgery, particularly for frail and vulnerable individuals.

Several limitations of this study must be acknowledged. The single-center, retrospective nature of the study inherently limits the ability to draw definitive causal conclusions. However, PSM was employed to mitigate the lack of randomization, ensuring balanced baseline characteristics between groups to strengthen the validity of the findings. Despite the use of PSM to minimize bias, residual unmeasured confounders, such as subtle aspects of frailty, nutritional status, or patient motivation may still have influenced the observed outcomes. The study benefits from a standardized anesthesiologic technique applied consistently across both general and NA, with a specialized, restricted group of experienced anesthesiologists performing all NA procedures. Additionally, all colorectal surgeries were performed by a single surgeon, further reducing procedural variability and allowing for a more focused analysis of outcomes. While the patients’ accrual appears adequate, the limited sample size after matching may have reduced the statistical power to detect differences in rare but clinically relevant events. Therefore, the absence of significant differences should be interpreted as a lack of evidence of a difference rather than evidence of no difference, acknowledging the potential for Type II error. The study clearly defines its outcomes of interest, which include measures such as pain, mental status, intraoperative surgical comfort, and postoperative complications. These outcomes are assessed using validated tools, enhancing the reliability of the data.

## 5. Conclusions

This study provides valuable insights into the application of NA in minimally invasive colorectal surgery in a population with specific inclusion criteria compared to a general population undergoing GA, demonstrating that NA is a feasible and effective alternative to GA. Our findings demonstrate that laparoscopic colorectal surgery can be performed using NA without affecting postoperative outcomes. Future prospective studies should include standardized PROMs to better capture subjective recovery trajectories and patient experience.

## Figures and Tables

**Table 1:** Demographic characteristics prior to PSM.

	GA (*n* = 47)	NA (*n* = 32)	TOTAL (*n* = 79)	*p* Value	Shapiro
**Age** Median (IQR)	72 (61–80)	83 (74–85)	75 (66–83)	*p* = *0.0005*	*p* = *0.0007*
**Gender** (Female)	25 (53.2%)	19 (59.4%)	44 (55.7%)	*p* = *0.6487*	
**BMI** kg/m^2^ Median (IQR)	24.2 (22.7–28)	25.4 (22.1–28.5)	25 (22–28)	*p* = *0.9681*	*p* < *0.0001*
**ASA**					
1	0 (0.0%)	1 (3.1%)	1 (1.3%)	*p* = *0.4922*	
2	28 (59.6%)	15 (46.9%)	43 (54.4%)		
3	17 (36.2%)	14 (43.7%)	31 (39.2%)		
4	2 (4.3%)	2 (6.3%)	4 (5.1%)		
**Prior abdominal surgery**	23 (49.0%)	19 (59.4%)	42 (53.2%)	*p* = *0.4912*	
**Diagnosis**					
Malignant	36 (74.5%)	28 (87.5%)	63 (79.7%)	*p* = *0.2538*	
Benign	12 (25.5%)	4 (12.5%)	16 (20.3%)		

ASA: American Society of Anesthesiologist Score; BMI: Body Mass Index; GA: general anesthesia; IQR: Interquartile Range; NA: neuraxial anesthesia.

**Table 2:** Demographic characteristics after PSM.

	GA (*n* = 32)	NA (*n* = 32)	TOTAL (*n* = 64)	*p* Value	Shapiro
**Age** Median (IQR)	82 (72–86)	83 (74–85)	82 (72.2–86)	*p* = *0.7466*	*p* < *0.0001*
**Gender** (Female)	23 (71.9%)	19 (59.4%)	44 (55.7%)	*p* = *0.4302*	
**BMI** kg/m^2^ Median (IQR)	25.5 (22.4–28.5)	25.4 (22.1–28.5)	25.5 (22.1–28.5)	*p* = *0.6049*	*p* = *0.0003*
**ASA**					
1	0 (0.0%)	1 (3.1%)	1 (1.3%)	*p* = *0.6876*	
2	12 (37.5%)	15 (46.9%)	27 (42.2%)		
3	17 (53.1%)	14 (43.7%)	31 (48.4%)		
4	3 (9.4%)	2 (6.3%	5 (7.8%)		
**Prior abdominal surgery**	21 (65.6%)	19 (59.4%)	40 (62.5%)	*p* = *0.7966*	
**Diagnosis**					
Malignant	29 (90.6%)	28 (87.5%)	57 (89.1%)	*p* = *1.0000*	
Benign	3 (9.4%)	4 (12.5%)	7 (10.9%)		

ASA: American Society of Anesthesiologist Score; BMI: Body Mass Index; GA: general anesthesia; IQR: Interquartile Range; NA: neuraxial anesthesia.

**Table 3:** Intraoperative outcomes after PSM.

	GA (*n* = 32)	NA (*n* = 32)	TOTAL (*n* = 64)	*p* Value	Shapiro
**Laparoscopic surgery**	32 (100%)	32 (100%)	64 (100%)	*p* = *1.000*	
**Primary procedure**					
Right colectomy with CME	23 (71.9%)	19 (59.4%)	42 (65.6%)	*p* = *0.3757*	
Left colectomy	6 (18.7%)	8 (25.0%)	14 (21.9%)		
Transverse resection	1 (3.1%)	0 (0.0%)	1 (1.6%)		
Low rectal anterior resection	2 (6.2%)	5 (15.6%)	7 (10.9%)		
**Stoma creation**	3 (9.4%)	6 (18.7%)	9 (14.1%)	*p* = *0.4741*	
**Anesthesia preparation** (min) Median (IQR)	30 (16.2–62.2)	42.5 (30–68)	40 (25.7–65)	*p* = *0.0115*	*p* = *0.0012*
**Total surgery duration** (min) Median (IQR)	231 (179–240)	181 (157–210)	200 (170–140)	*p* = *0.0089*	*p* = *0.0553*
**Drain**	8 (25.0%)	7 (21.9%)	15 (23.44%)	*p* = *1.000*	
**Conversion to open**	3 (9.4%)	0 (0.0%)	3 (4.7%)	*p* = *0.2381*	
**Associated procedure**	14 (43.7%)	10 (31.2%)	24 (37.5%)	*p* = *0.4390*	
**Fluid volume infusion** (cc) Median (IQR)	2000 (1525–2500)	1725 (1400–2100)	2000 (1500–2100)	*p* = *0.0805*	*p* < *0.0001*

CME: complete mesocolic excision; GA: general anesthesia; IQR: Interquartile Range; NA: neuraxial anesthesia.

**Table 4:** Postoperative outcomes after PSM.

	GA (*n* = 32)	NA (*n* = 32)	TOTAL (*n* = 64)	*p* Value	Shapiro
**ICU**	4 (12.5%)	2 (6.2%)	6 (9.4%)	*p* = *0.6719*	
**LOS** (days) Median (IQR)	4 (3–7)	5 (4–6)	4 (3–5)	*p* = *0.6360*	
**Leak**	0 (0.0%)	2 (6.2%)	2 (3.1%)	*p* = *0.4921*	
**Leak GRADE**					
A	0 (0.0%)	1 (3.1%)	1 (3.1%)	*p* = *1.000*	
B	0 (0.0%)	1 (3.1%)	1 (3.1%)		
C	0 (0.0%)	0 (0.0%)	0 (0.0%)		
**Complications** (overall)	12 (37.5%)	12 (37.5%)	24 (37.5%)	*p* = *1.0000*	
**Clavien–Dindo**					
0	20 (62.5%)	21 (65.6%)	41 (64.1%)	*p* = *0.2213*	
1	8 (25.0%)	3 (9.4%)	11 (17.2%)		
2	4 (12.5%)	7 (21.9%)	11 (17.2%)		
3a	0 (0.0%)	0 (0.0%)	0 (0.0%)		
3b	0 (0.0%)	0 (0.0%)	0 (0.0%)		
4a	0 (0.0%)	0 (0.0%)	0 (0.0%)		
4b	0 (0.0%)	0 (0.0%)	0 (0.0%)		
5	0 (0.0%)	1 (3.1%)	1 (3.1%)		
**Readmission** (<90 days)	0 (0.0%)	3 (9.4%)	3 (4.7%)	*p* = *0.2381*	

ICU: Intensive Care Unit; GA: general anesthesia; IQR: Interquartile Range; LOS: Length of Stay; NA: neuraxial anesthesia.

## Data Availability

The dataset used during the current study is available from the corresponding author upon reasonable request.

## Abbreviations

The following abbreviations are used in this manuscript:

ARISCAT	Assess Respiratory Risk in Surgical Patients in Catalonia (score)
CME	Complete Mesocolic Excision
CSE	Combined Spinal–Epidural (anesthesia)
ERAS^®^	Enhanced Recovery After Surgery
FiO_2_	Fraction of Inspired Oxygen
GA	General Anesthesia
IMA	Inferior Mesenteric Artery
IQR	Interquartile Range
LOS	Length of Stay
NA	Neuraxial Anesthesia
NSAIDs	Non-Steroidal Anti-Inflammatory Drugs
PEEP	Positive End-Expiratory Pressure
POCD	Postoperative Cognitive Decline
POD	Postoperative Day
PONV	Postoperative Nausea and Vomiting
PSM	Propensity Score Matching
PTSD	Post-Traumatic Stress Disorder
SD	Standard Deviation
SpO_2_	Peripheral Oxygen Saturation
TCI	Target Control Infusion
TV	Tidal Volume
